# Study of the sociodemographic, clinical and criminological characteristics of Tunisian female offenders

**DOI:** 10.1192/j.eurpsy.2024.1211

**Published:** 2024-08-27

**Authors:** N. Smaoui, O. Bouattour, I. Gassara, R. Feki, M. Bou Ali Maalej, J. Ben Thabet, L. Zouari, M. Maalej, N. Charfi, S. Omri

**Affiliations:** ^1^Psychiatry C department, Hedi Chaker university hospital; ^2^Faculty of Medicine, Sfax; ^3^University of Sfax, SFAX, Tunisia

## Abstract

**Introduction:**

The psychopathology of female crime perpetrators is not well understood since female criminality rates have remained distinctly lower than male criminality.

This study draws on over 20 years of psychiatric expertises to identify sociodemographic, clinical, and forensic characteristics of female perpetrators.

**Objectives:**

- To describe the epidemiological and clinical profile of female offenders examined for criminal psychiatric expertise.
- Describe the criminological and forensic characteristics of these women.

**Methods:**

Retrospective and descriptive study, which focused on 56 criminal psychiatric expertise files of female offenders, examined at the psychiatric department “C” at the CHU Hedi Chaker in Sfax, Tunisia, over a period of 24 years.

For each offender, we examined the expert report and the judicial research report. We then transcribed the socio-demographic and clinical information, as well as the criminological and forensic characteristics, onto a pre-established form.

**Results:**

The accused women in our study had an average age of 35 years and 06 months, and 67.86% of the cases were under 40 years of age, with an educational level no higher than primary school in 62.5% of cases. They were unemployed in 71.4% of cases. Among the accused examined, 76.8% had mental disorders, including 46.6% with personality disorders, 16.3% with intellectual disability, 16.3% with bipolar disorder, 9.3% with depressive disorder, 9.3% with psychotic disorder, and 2.3% with substance use disorder (anxiolytic). We recorded 55.4% offences against persons, including 37.5% homicides and attempted homicides, and 44.6% offences against property, including 23.2% thefts. Dementia in the legal sense was identified in 30.4% of cases. Bipolar disorder accounted for 41.1% of legally demented subjects.

**Conclusions:**

It emerges that the profile of the female criminal is that of a woman under 40, with a low educational and economic level, and most often with an antisocial personality or intellectual disability. It would therefore be important to step up primary prevention work by better educating these at-risk women and to combat the factors contributing to dangerousness among the mentally ill by optimizing their psychiatric care.

**Disclosure of Interest:**

None Declared

